# Aerobic capacity after lung resection in patients with COPD

**DOI:** 10.36416/1806-3756/e20240361

**Published:** 2025-07-22

**Authors:** Danielle C C Bedin, Roberta P Ramos, Elaine Brito Vieira, Ivan Ivanaga, Sonia M Faresin

**Affiliations:** 1. Grupo de Avaliação Pré-operatória. Disciplina de Pneumologia, Departamento de Medicina. Universidade Federal de São Paulo - UNIFESP - São Paulo (SP) Brasil.; 2. Unidade de Função Pulmonar e Fisiologia Clínica do Exercício. Disciplina de Pneumologia, Departamento de Medicina. Universidade Federal de São Paulo - UNIFESP - São Paulo (SP) Brasil.

## TO THE EDITOR:

Cardiopulmonary exercise testing is an important functional tool for the preoperative assessment of operability in lung resection for NSCLC.^1^ Among the parameters evaluated, peak VO_2_ (VO_2peak_) has been reported to be a better predictor of postoperative complications and mortality than are resting pulmonary and cardiac function.^2^ Despite advances in preoperative assessment, the magnitude of functional changes after lung resection, particularly in patients with COPD, is still matter of debate.^3^ ^,^ ^4^

We conducted a prospective study to quantify the impact of pulmonary resection on the exercise capacity of patients with COPD scheduled for lung resection surgery at our institution. All participants provided written informed consent, and the study was approved by the medical ethics committee of the institution (Reference no. 89661117.2.0000.5505).

Patients were divided into two groups: those with normal spirometry values (control group) and those with expiratory obstruction on baseline spirometry (COPD group).^5^

The preoperative assessment, which included functional analysis with spirometry, measurement of the DL_CO_, and cardiopulmonary exercise testing (CPET),^2^ was performed before and six months after the surgical procedure. Predicted postoperative FEV_1_, DL_CO_, and VO_2peak_ values were calculated at baseline according to segmental loss. All of the patients underwent lateral thoracotomy. For the analysis, continuous variables are presented as mean and standard deviation or as median and interquartile range, being compared by using the paired Student’s t-test, Wilcoxon test, or Mann-Whitney test, as appropriate. Two-way repeated-measures ANOVA was performed to compare functional changes between the two groups. Categorical variables are presented as absolute and relative frequencies and were compared by using Fisher’s exact test. The Kolmogorov-Smirnoff test was employed to assess the normality of the distribution of all variables. Values of p < 0.05 were considered statistically significant. All analyses were performed with the IBM SPSS Statistics software package, version 19.0 (IBM Corporation, Armonk, NY, USA).

During the study period, 18 patients underwent preoperative assessment: 10 in the COPD group and 8 in the control group. The diagnosis of COPD was defined by the presence of clinical findings consistent with the disease, a history of smoking, and an FEV_1_/FVC ratio below the lower limit of reference. Baseline clinical characteristics and pulmonary function test results are shown in Table 1. The complication rates in the postoperative period were similar between the two groups. In the control group, 3 patients presented with respiratory infections, whereas in the COPD group, 3 patients presented with respiratory infections and 1 presented with atrial fibrillation (p = 0.91). Similarly, there were no differences between the two groups in terms of the length of ICU stay and hospital stay (Table 1). Because there were no deaths in either group during the study period, all of the patients in our sample underwent evaluation at baseline and postoperatively. It is also noteworthy that the extent of lung resection was comparable between the two groups (Table 1).

**Table 1:** Characteristics of patients in the control and COPD groups before and six months after lung resection.

Variables	Control			COPD		
	(n = 8)			(n = 10)		
	preoperative	postoperative	p	preoperative	postoperative	p
Age (years), mean ± SD	66.0 ± 9.0			66.9 ± 9.4		0.84
Male sex, n (%)	6 (75)			6 (60)		0.64
Body mass index (kg/m^2^), mean ± SD	24.5 ± 3.2			23.7 ± 3.0		0.63
Smoking status, n %						
Never smoker	0 (0)			0 (0)		
Former smoker	6 (75)			3 (30)		0.057
Current smoker	2 (25)			7 (70)		
Resected segments (n), median [IQR]	4 [2-5]			3 [3-5]		0.90
Hospital stay (days), median [IQR]	6 [5-7]			7 [5-9]		0.90
ICU stay (days), median [IQR]	3 [2-6]			4 [3-5]		0.46
Spirometry parameters, mean ± SD						
FEV_1_ (%)	86.1 ± 16.4	80.6 ± 17.7	0.40	65.4 ± 10.1	62.1 ± 14.5	0.46
FEV_1_/FVC ratio	0.75 ± 0.03	0.76 ± 0.03	0.33	0.60 ± 0.06	0.60 ± 0.08	0.08
DL_CO_ (%)	73.8 ± 15.9	63.2 ± 14.6	0.13	67.5 ± 22.6	53.6 ± 19.6	0.09
CPET parameters, mean ± SD or median [IQR]						
Work load, watts	94.6 ± 33.3	82.0 ± 31.5	0.005	69.5 ± 23.3	59.9 ± 25.8	0.01
VO_2peak_, mL/min	1350 ± 438.0	1108.4 ± 320.0	0.004	993.9 ± 240.0	937.0 ± 30.0	0.16
VO_2peak_, mL/kg/min	20.8 ± 7.1	16.3 ± 5.3	0.007	15.6 ± 3.7	14.5 ± 4.1	0.07
VO_2peak_, % of predicted	91.5 ± 18.9	77.4 ± 17.9	0.01	74.9 ± 16.6	70.5 ± 19.7	0.15
VO_2_ LT, %	55.9 ± 18.3	41.4 ± 7.9	0.14	49.3 ± 10.1	44.1 ± 10.2	0.36
DVO_2_/DW	10.3 ± 1.9	9.5 ± 2.7	0.10	8.9 ± 2,3	10.9 ± 1,7	0.02
VE _peak_, L/min	62.0 ± 19.2	49.8 ± 10.2	0.06	43.8 ± 10.2	48.9 ± 8.4	0.66
VE /MVV_peak_	0.55 ± 0.16	0.52 ± 0.09	0.74	0.53 ± 0.1	0.6 ± 0.2	0.38
VE/VCO_2peak_	31.8 ± 4.3	32.4 ± 3.4	0.38	31.4 ± 4.0	31.6 ± 4.3	0.87
DVE/DVCO_2peak_	35.1 ± 5.6	36.6 ± 5.5	0.42	34.0 ± 4.7	34.8 ± 5.6	0.56
Intercept DVE/DVCO_2VT_, L/min	+2.7 ± 2.1	3.0 ± 1.9	0.93	2.9 ± 2.0	4.4 ± 2.4	0.04
Intercept DVE/DVCO_2peak_, L/min	+0.9 ± 2.5	0.5 ± 2.8	0.79	1.5 ± 2.0	2.3 ± 2.9	0.07
Nadir VE/VCO_2peak,_ L/min	34.3 ± 2.7	36.6 ± 3.3	0.08	35.7 ± 4.1	37.8 ± 3.9	0.11
HR_peak_, bpm	145 ± 16	134 ± 13	0.001	120 ± 17	114 ± 22	0.69
VO_2_/HR_peak_, % of predicted	102.8 ± 26.7	92.4 ± 25.6	0.12	100.8 ± 18.3	103.2 ± 24.6	0.83
DHR/DVO_2_, beats/L	62 ± 18	61 ± 12	0.91	56.7 ± 22.1	47.6 ± 16.2	0.40
SpO_2_ at rest, %	98 [97-99]	97 [97-98]	0.34	97 [95-97]	96 [94-97]	0.72
Peak SpO_2_, %	98 [96-99]	97 [97-99]	0.32	97 [90-98]	92 [88-96]	0.11

VO_2peak_: peak VO_2_; VO_2_ LT: VO_2_ at the lactate threshold; W: work rate; VE_peak_: peak VE; MVV: maximal voluntary ventilation: V*C*O_2_: carbon dioxide output; and VT: ventilatory *threshold*.

There were no significant differences between the preoperative (baseline) and postoperative (month-six) time points, in either group, in terms of the spirometric and DL_CO_ values (Table 1). However, the pattern of change in VO_2peak_ (in absolute values and adjusted for body weight) was significantly different between the two groups, being significantly greater postoperatively in the control group (Table 1). These findings were reinforced by the ANOVA, which demonstrated a distinct behavior of VO_2peak_ between the two groups and time points (p = 0.011 and p = 0.024, respectively, for the absolute and adjusted values). In addition, the decrease in VO_2peak_ relative to the number of resected segments was greater in the control group than in the COPD group, with a median loss of 60.2 mL/min/segment in the control group and 20.3 mL/min/segment in the COPD group (p = 0.043). In the COPD group, predicted VO_2peak_ values underestimated the actual values obtained six months after the surgery, whereas no significant difference was observed in the control group (Table 2).

**Table 2:** Comparison between the predicted and actual postoperative peak VO_2_ values at six months after lung resection.

Variable	Control		p	COPD		p
	(n = 8)			(n = 10)		
	Predicted	Actual		Predicted	Actual	
VO_2peak_, mL/min	1105.0 ± 399.4	1108.4 ± 20.0	0.96	815.1± 233.2	937.0 ± 306.0	0.007
VO_2peak_,% pred	73.6 ± 17.8	77.4 ± 17.9	0.54	60.9 ± 15.8	70.5 ± 19.7	0.003
VO_2peak_,mL/kg/min	16.9 ± 5.9	16.3 ± 5.3	0.68	12.8 ± 3.6	14.5 ± 4.1	0.007

VO_2peak_: peak VO_2_.

In our study sample, the patients with COPD presented no significant deterioration of VO_2peak_ after lung resection, whereas the patients with preserved lung function at baseline presented greater declines, not only in absolute values but also in VO_2peak_ per resected segment. In addition, the predicted values of FEV_1_ and VO_2peak_ overestimated the functional deterioration in the patients with COPD.

In a study of 12 patients with COPD, Bobbio et al.^6^ found a 21% decrease in VO_2peak_ at three months after lung resection. Miyoshi et al. also observed a decrease in VO_2peak_ after lung resection, although without differentiating between patients with COPD and patients with preserved lung function at baseline. In addition, those authors revaluated exercise capacity after a shorter follow-up period. Similarly, Bolliger et al.^1^ showed that the VO_2peak_ decreased by three months after the operation but reverted to the preoperative values by six months after, without specifically addressing patients with COPD. That finding aligns with the minor changes in lung function observed in our study at six months after the surgical procedure. 

In parallel with the pulmonary function tests, predicted postoperative VO_2peak_ was similar to the actual measurements six months after surgery in the control group but was significantly lower than the actual measurements in the COPD group. These findings are consistent with those of Brunelli et al.,^8^ who noted the imprecision of predicted postoperative VO_2_, particularly when the preoperative values are lower.

We find it interesting that, after the VO_2peak_ decrease had been adjusted for the number of resected segments, the functional loss was three times greater in our control group than in our COPD group (60.2 mL/min/segment vs. 20.3 mL/min/segment). Previous studies of patients undergoing segmentectomy, lobectomy, or pneumonectomy have shown that greater lung tissue loss results in greater loss of pulmonary function.^1^ ^,^ ^9^ However, the authors of those studies did not take the baseline ventilatory pattern or the presence of COPD into account. 

Our study has limitations that should be acknowledged. It was a single-center study conducted by the same team of thoracic surgeons and pulmonologists in order to ensure uniformity in the preoperative evaluation. The postoperative tests were performed six months after surgery, a time point chosen somewhat arbitrarily on the basis of data in the literature suggesting that lung function stabilizes at three to six months after pulmonary resection.^4^ ^,^ ^10^ Our results show that patients with COPD submitted to lung resection demonstrated distinct functional behavior. However, the limitations imposed by our sample size should be considered before our findings are extrapolated to other populations. In addition, our COPD group included only patients with mild or moderate COPD, which might preclude extrapolation of our results to patients with severe COPD. However, the fact that such patients were not included allowed us to speculate that the lack of room for deterioration after surgery did not explain the small size of the decrease in functional capacity in the COPD group. Furthermore, we did not correlate the functional findings with patient reported outcome measures, which could have reinforced the relevance of our findings.

In our study, the predicted postoperative VO_2peak_ values appeared to overestimate the decrease in aerobic capacity following lung resection in patients with mild or moderate COPD. Our findings suggest that, in patients with COPD, there is a need for a more comprehensive preoperative analysis, with less emphasis on the predicted postoperative CPET values.
